# Change in the Cortical Complexity of Spinocerebellar Ataxia Type 3 Appears Earlier than Clinical Symptoms

**DOI:** 10.1371/journal.pone.0118828

**Published:** 2015-04-21

**Authors:** Tzu-Yun Wang, Chii-Wen Jao, Bing-Wen Soong, Hsiu-Mei Wu, Kuo-Kai Shyu, Po-Shan Wang, Yu-Te Wu

**Affiliations:** 1 Department of Biomedical Imaging and Radiological Sciences, National Yang-Ming University, Taipei, Taiwan, ROC; 2 Department of Recreation Sports and Health Promotion, Asia-Pacific Institute of Creativity, Tao-Fen, Taiwan, ROC; 3 The Neurological Institute, Taipei Veterans General Hospital, Taipei, Taiwan, ROC; 4 Department of Neurology, National Yang-Ming University School of Medicine, Taipei, Taiwan, ROC; 5 Department of Radiology, Taipei Veterans General Hospital, Taipei, Taiwan, ROC; 6 Department of Electrical Engineering, National Central University, Chung-Li, Taiwan, ROC; 7 The Neurological Institute, Taipei Municipal Gan-Dau Hospital, Taipei, Taiwan, ROC; 8 Institute of Biophotonics, National Yang-Ming University, Taipei, Taiwan, ROC; University of Texas MD Anderson Cancer Center, UNITED STATES

## Abstract

Patients with spinocerebellar ataxia type 3 (SCA3) have exhibited cerebral cortical involvement and various mental deficits in previous studies. Clinically, conventional measurements, such as the Mini-Mental State Examination (MMSE) and electroencephalography (EEG), are insensitive to cerebral cortical involvement and mental deficits associated with SCA3, particularly at the early stage of the disease. We applied a three-dimensional fractal dimension (3D-FD) method, which can be used to quantify the shape complexity of cortical folding, in assessing cortical degeneration. We evaluated 48 genetically confirmed SCA3 patients by employing clinical scales and magnetic resonance imaging and using 50 healthy participants as a control group. According to the Scale for the Assessment and Rating of Ataxia (SARA), the SCA3 patients were diagnosed with cortical dysfunction in the cerebellar cortex; however, no significant difference in the cerebral cortex was observed according to the patients’ MMSE ratings. Using the 3D-FD method, we determined that cortical involvement was more extensive than involvement of traditional olivopontocerebellar regions and the corticocerebellar system. Moreover, the significant correlation between decreased 3D-FD values and disease duration may indicate atrophy of the cerebellar cortex and cerebral cortex in SCA3 patients. The change of the cerebral complexity in the SCA3 patients can be detected throughout the disease duration, especially it becomes substantial at the late stage of the disease. Furthermore, we determined that atrophy of the cerebral cortex may occur earlier than changes in MMSE scores and EEG signals.

## Introduction

Spinocerebellar ataxia type 3 (SCA3) is a hereditary neurodegenerative disorder caused by CAG expansion in the coding region of chromosome 14q32.1 [1, 2]. Clinically, SCA3 patients are characterized by prominent and progressive cerebellar ataxia combined with degeneration of the cerebellum and its afferent and efferent connections [3]. Recent studies have indicated that SCA3 patients exhibit mental deficits, such as cognitive impairment in executive functions, verbal and visual memory, visuoconstruction, visual attention, and emotional deficits [4–6].

Currently, an increasing amount of evidence has indicated the involvement of the cerebral cortex in SCA3 [7, 8]. Mutant ataxin-3 proteins have been observed in cerebral cortex and could contribute to the degeneration of nerve cells [9]. Moreover, subclinical abnormalities in the cerebral cortex have been identified in functional [10, 11] and neuropathological studies [12]. Voxel-based morphometry (VBM) have indicated involvement of various regions of the cerebrum, including the frontal and temporal lobes [13]; temporal gyrus, bilateral inferior temporal gyrus, and cingulate gyrus [14]; putamen, cingulum, precentral, and parietal lobe [15]; and bilateral putamen and pallidum [16]. However, the sites of cerebral involvement in SCA3 were inconsistent in these VBM studies [17, 18].

Measuring the cortical shape can provide an alternative to the volumetric method for assessing morphological changes in the brain. Fractal analysis, gyrification index and curvedness have been proposed to quantify the structural complexity of the cortex and select abnormalities in the cortical structure in patients with movement disorders [19–31]. Fractal analysis has shown decreased cortical complexity in patients with mental disorders comparing to normal controls [32–35]. In addition, fractal analysis has been used to investigate relationship between cortical complexity and intelligence as well as cognitive ability [36–38].

In SCA3 patients, motor-related dysfunction has generally been assessed using the SARA [39]; however, no reliable and easily implemented measure of mental decline in SCA3 patients has been developed. The extracerebellar symptoms of SCA3 were recently assessed using the Inventory of Non-Ataxia Symptoms (INAS) [40]. However, the INAS score is a dimensionless value and cannot be used to measure the severity of SCA3 [16]. Previous studies have shown that the degree of cortical degeneration, which was quantified using fractal dimension (FD) analysis, can indicate the severity of neurodegenerative disorders [22, 23]. A FD analysis that condenses all of the structural details of an irregular object into a single numeric value is advantageous for producing results with smaller standard deviations and sex effects compared with the traditional volumetric method [21, 23]. This method can be used for effectively measuring structural cortical changes in both the cerebellar and extracerebellar regions and facilitated evaluating the severity of the cortical atrophy in this SCA3 study.

In addition, researchers have delineated the disease progression of cortical dysfunction in SCA3 patients [3, 16, 41, 42]. However, few studies have reported the association between the disease progression of cerebral dysfunction and cortical abnormality in SCA3 patients. D'Abreu, Franca, et al. (2012) could not determine the progression of atrophy within a short duration by using magnetic resonance spectroscopy, and Reetz, Costa, et al. (2013) reported that disease progression was limited in the putamen and caudate nucleus, in addition to cerebellum [16, 42].

In this study, we combined the clinical features of SCA3 and the FD method to (1) identify the pattern of affected regions in the cortex of SCA3 patients by measuring the regional cortical changes that occur in supratentorial regions, (2) distinguish whether cortical complexity is associated with the clinical symptoms, such as MMSE and EEG signals, observed in SCA3, and (3) investigate the correlation between the progression of cerebral dysfunction and cerebellar dysfunction.

## Materials and Methods

### 1. Participants

The Institutional Review Board of Taipei Veterans General Hospital approved this study. All participants provided written informed consent before participating. Forty-eight SCA3 patients were recruited from 2005 to 2012. A group of 50 healthy volunteers were matched according to age, sex, and years of education, serving as a control group. An ataxial clinical assessment of all of the patients was conducted using SARA scores and modified Hoehn and Yahr staging [43]. In addition, 17 patients received routine electroencephalography (EEG) examinations and 14 patients were assessed using the MMSE [44]. Table 1 shows the demographic, clinical, and MRI data for both groups. SCA3 patients who experienced progressive and otherwise unexplained ataxia and tested positive for the SCA type 3 genotype were included in this study. The disease duration of the SCA3 patients was 8.89 ± 6.432 years. To further investigate the difference in cortical change according to disease duration, we separated the SCA3 patients into two groups: an early stage group (23 patients of whom the disease duration was shorter than 8 years) and a late stage group (25 patients of whom the disease duration was longer than 8 years).

**Table 1:** The demographic, clinical, and MR image data of control and patient groups.

*Characteristic*	*Group*		
	Controls (N = 50)	SCA3 Patients (N = 48)	*p* value
**Sex(F/M)**	25/25	21/27	0.535^a^
**Age(years)** ^**†**^	48.24 ± 13.956	48.13 ± 11.747	0.516^b^
**Duration(years)** ^**†**^	—	8.89 ± 6.432	—
**SARA** ^**†**^	—	14 ± 8.103	—
**H & Y staging** ^**†**^	—	2.88 ± 1.19	—
**MMSE** ^**†**^	—	28.5 ± 1.61	—
**EEG signals observation(abnormal/normal)**	—	5/12	—
**Cerebral atrophy observed via visual inspection**	—	9	—
**Cerebellar atrophy observed via visual inspection**	—	39	—

SARA = Scale for the Assessment and Rating of Ataxia; MMSE = Mini-Mental State Examination; H & Y staging = Hoehn and Yahr Staging Scale

^a^ Pearson's chi square test(*χ* ^2^ = 0.384);

^b^ two-tailed two-sample *t*-test;

^†^ Continuous variables are expressed as mean ± standard deviation (SD);

The members of the control group had no central nervous system disease and exhibited no neurological abnormalities during the study period. An experienced neuroradiologist examined the T1- and T2-weighted images of the control group to ensure the absence of latent neurological diseases or unexpected abnormalities.

### 2. Electroencephalography

#### 2.1 Recording

The EEG data were collected when the participants were sitting relaxed and with their eyes closed. Alertness was continually monitored to prevent drowsiness. The EEGs were recorded using a 19-channel digital portable EEG machine (Nicolet Biomedical Inc., Madison, WI) for 5 minutes. All electrodes were placed based on the configuration of the international 10–20 system with a linked ear reference (mean EEG signal of electrodes at bilateral ears). Electrode skin impedance was usually less than 3 kΩ. The signals were digitized at a rate of 256 Hz by using a 12-bit analogue-to-digital converter and processed using an analogous band-pass filter (0.05−70 Hz) and a 60-Hz notch filter.

#### 2.2 Criteria of the abnormality in EEG

Abnormal EEG patterns of the SCA3 patients were identified by experienced neurologists, which included spikes, intermittent slow activity, diffuse background slowing and frontal and occipital intermittent rhythmic delta activity. Experienced neurologists examined all the EEG recordings without prior informing that these recordings collected from SCA3 patients or other participants.

### 3. Magnetic resonance imaging scan

#### 3.1 Data acquisition

Axial MR brain images encompassing the entire cerebrum and cerebellum were acquired using a 1.5-T Vision Siemens scanner (Erlangen, Germany). The participants were scanned using a circularly polarized head coil to obtain the T1-weighted images (TR, 14.4 ms; TE, 5.5 ms; matrix size: 256 × 256; 1.5-mm axial slices; FOV = 256 mm × 256 mm; voxel size, 1.0 × 1.0 × 1.5 mm).

#### 3.2 Data processing

**Fig 1:**
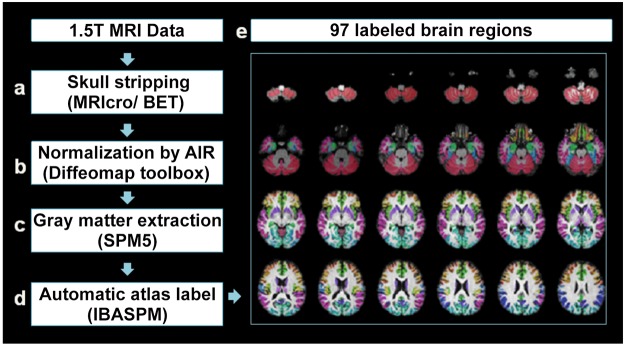
Procedure for image preprocessing. Each voxel of gray matter in every T1-weighted image was anatomically assigned to the resulting 97 labeled brain regions, which were displayed in different colors in the lower right panel.

The acquired T1-weighted image of each participant was reformatted into an axial image and converted to the Analyze format by using MRIcro software (Chris Rorden, University of Nottingham, UK; www.sph.sc.edu/comd/rorden/mricro.html). Fig 1 shows the data-processing flowchart. To improve the accuracy of brain tissue extraction, an automatic skull-stripping function was applied to the image volumes by using the brain extraction tool in the MRIcro software (Fig 1a). Fig 1b shows the normalization process, which was conducted using DiffeoMap (Li, X.; Jiang, H.; and Mori, S.; Johns Hopkins University, www.MriStudio.org). In this procedure, a 12-parameter affine transformation (AIR; Woods, Grafton [45]) was used to normalize each T1-weighted image toward the JHU_MNI_SS_T_ss T1 template.

#### 3.3 Atlas extraction

**Fig 2:**
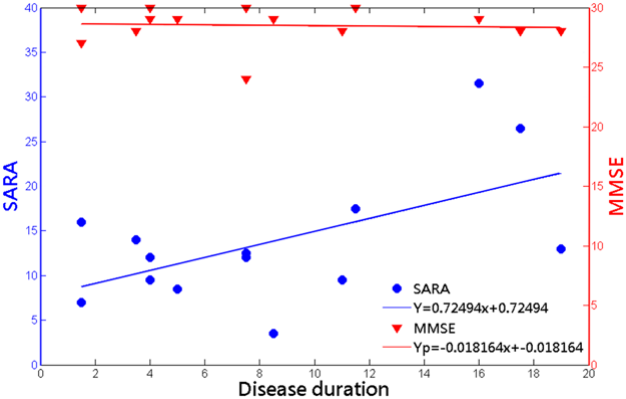
The cortical dysfunction of SCA3 patients in cerebellar cortex (SARA; left vertical axis) and in cerebral cortex (MMSE; reight vertical axis) were incoherent. The result shows significantly increasing SARA scores throughout duration of disease (*r* = 0.5672; *p* = 0.0344), but nonsignificant correlation between MMSE scores and duration of disease (*r* = -0.2138; *p* = 0.4100). (SARA: Y = 0.72494x+7.6755; MMSE: Y*p* = -0.0182x+28.6531).

Image data processing was conducted using the SPM5 toolbox (Welcome Department of Cognitive Neurology, Institute of Neurology, University College London, London, UK, http://www.fil.ion.ucl.ac.uk/spm/) and the Individual Brain Atlases using Statistical Parametric Mapping (IBASPM; http://www.thomaskoenig.ch/Lester/ibaspm.htm) toolbox in MATLAB 7.0 software (Mathworks, Natick, MA, USA). This process consisted of the following steps: (1) the normalized image volume was segmented into gray matter, white matter, and cerebral spinal fluid in native space (Fig 1c), and (2) the gray matter image was transformed into Montreal Neurological Institute space [46], and each voxel of gray matter was anatomically assigned to one of the 116 automatic anatomical label structures by using IBASPM [46] (Fig 1d). The 26 regions of the cerebellum were then merged into 7 regions according to their anatomical structures, and the volumes of 97 labeled (45 for each cerebral hemisphere; Table 2) brain structures were extracted for each participant (Fig 1e).

**Table 2:** Cortical and sub-cortical regions defined in Automated Anatomical Labeling template image in standard stereotaxic space (Each region is divide into left and right).

Region name	Abbreviation	Region name	Abbreviation
Precentral gyrus	PreCG	Lingual gyrus	LIN
Superior frontal gyrus	SFG	Superior occipital gyrus	SOG
Orbitofrontal cortex (superior)	ORBsup	Middle occipital gyrus	MOG
Middle frontal gyrus	MFG	Inferior occipital gyrus	IOG
Orbitofrontal cortex (superior-medial)	ORBsupmed	Fusiform gyrus	FUG
Inferior frontal gyrus (opercular)	IFGoper	Postcentral gyrus	PostCG
Inferior frontal gyrus (triangular)	IFGtraing	Superior parietal gyrus	SPG
Orbitofrontal cortex (inferior)	ORBinf	Inferior parietal gyrus	IPG
Rolandic operculum	ROL	Supramarginal gyrus	SM
Supplementary motor area	SMA	Angular gyrus	ANG
Olfactory cortex	OLF	Precuneus	PCUN
Superior frontal gyrus (medial)	SFGmed	Paracentral lobule	PL
Orbitofrontal cortex (middle)	ORBmid	Caudate nucleus	CAU
Gyrus rectus	REG	Lenticular nucleus, putamen	PUT
Insula	INS	Lenticular nucleus, pallidum	PAL
Anterior cingulate gyrus	ACC	Thalamus	THA
Middle cingulate gyrus	MCC	Heschl gyrus	HES
Posterior cingulate gyrus	PCC	Superior temporal gyrus	STG
Hippocampus	HIP	Temporal pole (superior)	TPOsup
Parahippocampal gyrus	PHIP	Middle temporal gyrus	MTG
Amygdala	AMY	Temporal pole (middle)	TPOmid
Calcarine fissure and surrounding cortex	CAL	Inferior temporal gyrus	ITG
Cuneus	CUN		

#### 3.4 Three-dimensional box-counting method for fractal analysis

Mandelbrot originally proposed fractal analysis to quantify the shape complexity of objects into a single numerical value [47]. Because fractal analysis involves topologically measuring complexity, a high FD value represents a high degree of topological complexity in the examined tissue [48]. In this study, we adopted the 3D box-counting method because it can be used to evaluate the FD of structures with or without self-similarity [26, 48]. Thus, we obtained the 3D-FD values of 97 regions of gray matter from the entire brain for each participant. The algorithm of box-counting method was illustrated in our previous studies [22].

### 4. Statistical analysis

The normality of each data group was verified using the Jarque-Bera test [49]. The sex and age differences between the groups were measured using the Pearson chi-square test (*χ* ^2^ = 0.384, *p* = 0.535) and the 2-tailed 2-sample *t* test (*p* = 0.516), respectively.

We used Pearson’s *r* measurement to investigate the correlations between disease duration and SARA scores and between disease duration and MMSE scores. The 2-tailed *t* test was used to determine whether the 3D-FD values of each cortical region differed significantly between the control group and the SCA3 group. The significance of the results was corrected according to the false discovery rate (threshold of 0.05) [50].

The magnitude of the association between the 3D-FD value of individual brain regions and clinical features, such as disease duration, SARA scores, and MMSE scores, was determined using Pearson’s *r* measurement. We conducted multiple linear regressions to verify the association between disease duration and 3D-FD values (as a dependent variable), with age controlled. In addition, we explored the differences in the progression rate in each parcellated region of the cerebral cortex by using an ANOVA (with a Bonferroni test for post hoc analyses). These analyses were conducted using the Statistics Toolbox in MATLAB 7.0.

## Results

### SCA3 patients exhibited cortical dysfunction in the cerebellar cortex (SARA), but no significant difference in the cerebral cortex (MMSE)

Fig 2 shows the variation in the SARA and MMSE scores of 14 SCA3 patients with respect to the duration of disease. The results indicated that the SARA scores of SCA3 patients significantly increased as the duration of the disease increased (*r* = 0.5672; *p* = 0.0344). The SCA3 patients exhibited no correlation between MMSE scores and disease duration (*r* = -0.2138; *p* = 0.4100), and only one of the SCA3 patients met the criteria for dementia (MMSE < 26).

In addition, 5 of 17 SCA3 patients exhibited abnormal EEG signals in the cerebral hemisphere. Specifically, 3 of 5 SCA3 patients with abnormal EEG recording show intermittent slowing, and 2 of 5 SCA3 patients with abnormal EEG recording exhibited diffuse background slowing. We accordingly classified the SCA3 patients into 2 groups: SCA3 patients with abnormal EEG signals (5 cases) and SCA3 patients with normal EEG signals (12 cases). The disease durations of SCA3 patients with abnormal EEG signals has the trend to be longer than that of SCA3 patients with normal EEG signals (duration for SCA3 patients with abnormal EEG signals: 13.3 ± 5.57; duration for SCA3 patients with normal EEG signals: 6.71 ± 3.51).

### Significant correlation between decreased 3D-FD values and disease duration in SCA3

**Fig 3:**
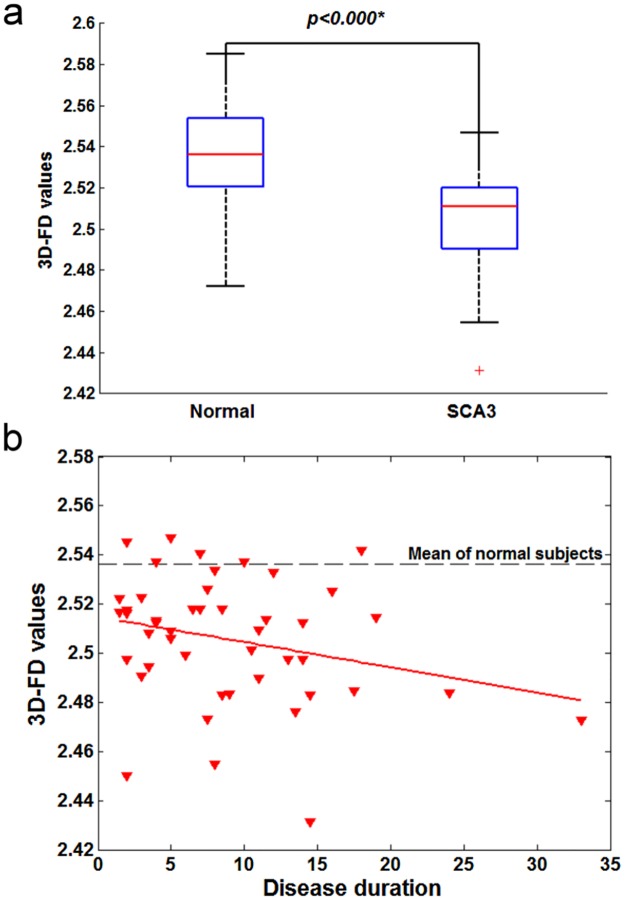
Cerebral cortex of SCA3 patients. (a) significant decrease in 3D-FD values in comparison with that of normal subjects. (b) A significant correlation was observed between decreased 3D-FD values and disease duration in cerebral cortex of SCA3 (r = -0.0330, p = 0.0287).

**Fig 4:**
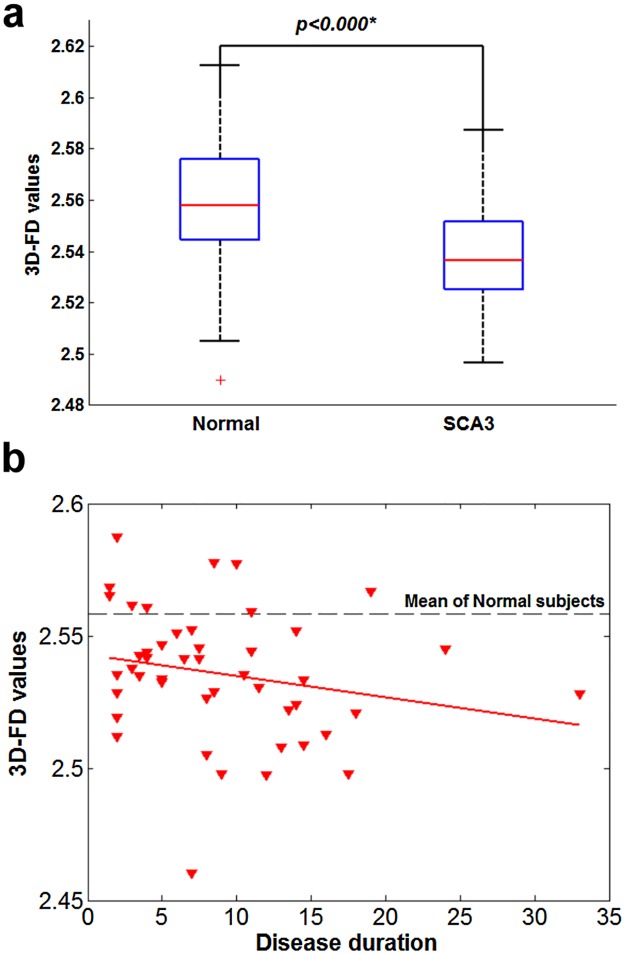
Cerebellar cortex of SCA3 patients. (a) significant decrease in 3D-FD values in comparison with that of normal subjects. (b) A significant correlation was observed between decreased 3D-FD values and disease duration in cerebellar cortex of SCA3 (*r* = -0.0318, *p* = 0.0354).

The 3D-FD values of the cerebellar cortex and cerebral cortex of the SCA3 patients exhibited significant decreases compared with those of the control participants (Fig 3a and Fig 4a). We observed a significant correlation between decreased 3D-FD values of both the cerebral and cerebellar cortices and disease duration in the SCA3 patients (Fig 3b and Fig 4b).

### Change of the cerebral cortex in SCA3 patients was more substantial at the late stage of the disease

**Fig 5:**
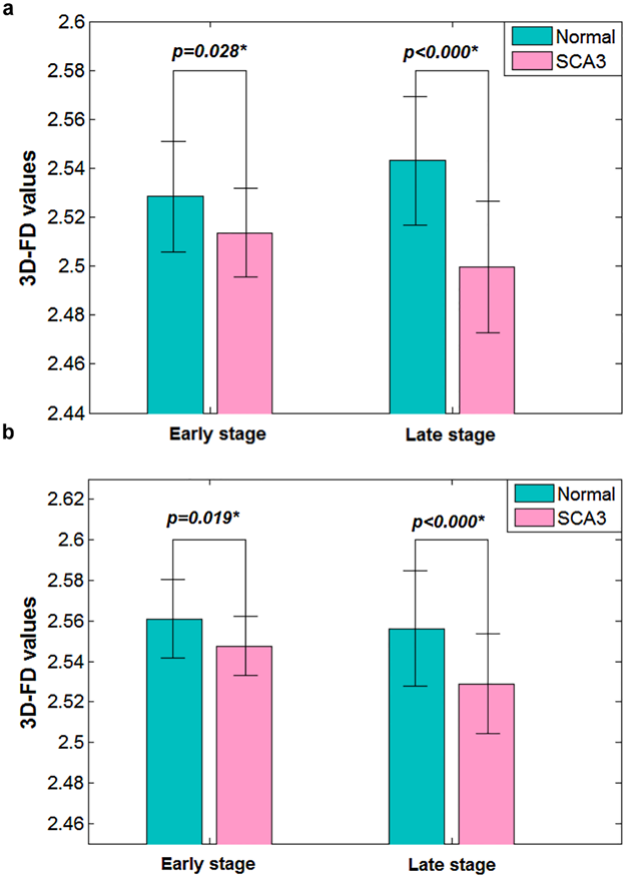
Significant differences of 3D-FD values between SCA3 patients and normal subjects at early stage and late stage of disease. (a) The cerebral cortex. (b) The cerebellar cortex. SCA3 shows significant decreased 3D-FD values at both the early and late stage of disease. Comparing the mean values of 3D-FD values at early stage of disease with that at late stage, the cortical atrophy in SCA3 patient was more substantial at the late stage of disease.

The SCA3 patients exhibited a significant correlation between the 3D-FD values of the cerebellar cortex and SARA scores (*r* = −0.3346; *p* = 0.023). However, the correlation between the 3D-FD values of the cerebral cortex and MMSE scores in the SCA3 patients was nonsignificant. When we used the 3D-FD method, the SCA3 patients exhibited significantly decreased FD values, even at the early stage. The mean 3D-FD values of the SCA3 patients at the early stage were greater than those at the late stage of the disease (Fig 5a and Fig 5b). This result suggested that cortical atrophy is more apparent at the late stage than at the early stage of the disease.

### Widespread involvement of the supratentorial regions in SCA3 patients

**Fig 6:**
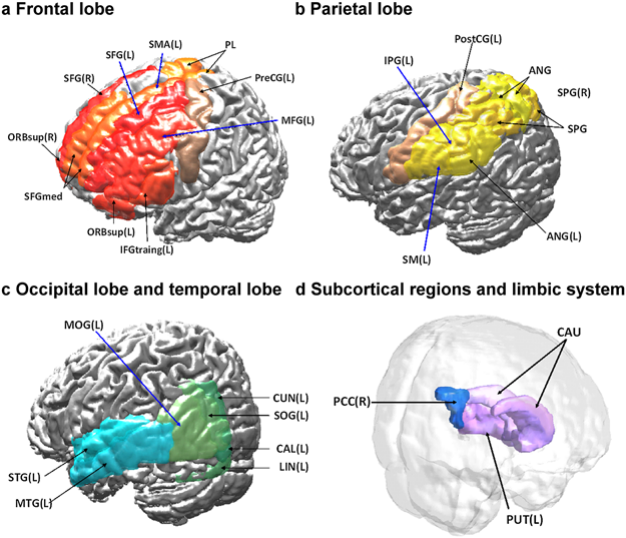
Widespread area in supratentorial regions show decreased 3D-FD value in SCA3 patients. (a, b) Frontal and parietal lobe of SCA3 exhibit decreased 3D-FD values, respectively. c, d Green, aqua-blue, violet and blue regions exhibit decreased 3D-FD value in the occipital lobe, temporal lobe, subcortical regions and limbic system, respectively.

3D-FD analysis indicated that 37 parcellated regions of the cerebral and cerebellar cortices exhibited significant regional variation in the SCA3 patients compared with the controls (Table 3). Among the parcellated regions, 32 were located in the cerebral cortex and 5 were located in the cerebellar cortex (Fig 6). Notably, the posterior lobe of the cerebellar cortex show significant decrease in SCA3 patients. In addition, unlike previous studies based on the volumetric method or postmortem analysis [51], our study revealed no significant difference in the 3D-FD values of the bilateral thalamus between the SCA3 patients and the healthy participants. We speculated that the thalamus may shrink but retain its shape, and that the topological complexity of an atrophied thalamus may not change. Consequently, atrophy of the thalamus might not be detected using the FD method.

**Table 3:** Brain regions with significant difference in 3D-FD values between normal control and SCA3 patients.

Region name	Side	Mean ± std	Mean ± std	*p*-value
*Cerebellar cortex*				
Entire cerebellar cortex	—	2.558 ± 0.024	2.531 ± 0.035	<0.001*
anterior_lobe of cerebellar cortex	L	2.168 ± 0.037	2.110 ± 0.069	<0.001*
	R	2.152 ± 0.04	2.030 ± 0.08	<0.001*
posterior_lobe_upper of cerebellar cortex	L	2.472 ± 0.025	2.452 ± 0.04	0.004*
	R	2.481 ± 0.03	2.441 ± 0.036	<0.001*
Vermis	—	2.154 ± 0.048	2.118 ± 0.043	<0.001*
*Cereberal cortex*				
Entire cerebral cortex	—	2.536 ± 0.024	2.506 ± 0.025	<0.001*
*Frontal lobe*				
Precentral gyrus	L	2.146 ± 0.066	2.067 ± 0.07	<0.001*
Superior frontal gyrus	L	2.078 ± 0.033	2.045 ± 0.051	<0.001*
	R	2.132 ± 0.04	2.099 ± 0.057	0.001*
Middle frontal gyrus	L	2.278 ± 0.035	2.247 ± 0.04	<0.001*
Orbitofrontal cortex (superior-medial)	L	2.109 ± 0.039	2.084 ± 0.054	0.009*
	R	2.138 ± 0.04	2.101 ± 0.047	<0.001*
Inferior frontal gyrus (opercular)	R	2.099 ± 0.05	2.067 ± 0.05	0.002*
Inferior frontal gyrus (triangular)	L	2.266 ± 0.042	2.229 ± 0.047	<0.001*
Supplementary motor area	L	2.187 ± 0.049	2.135 ± 0.047	<0.001*
Superior frontal gyrus (medial)	L	2.173 ± 0.041	2.124 ± 0.061	<0.001*
	R	2.087 ± 0.05	2.057 ± 0.067	0.014*
Paracentral lobule	L	2.044 ± 0.067	1.996 ± 0.075	0.001*
	R	1.978 ± 0.07	1.921 ± 0.08	<0.001*
*Parietal lobe*				
Postcentral gyrus	L	2.173 ± 0.054	2.100 ± 0.057	<0.001*
Superior parietal gyrus	L	2.095 ± 0.055	2.028 ± 0.063	<0.001*
	R	2.075 ± 0.06	2.038 ± 0.072	0.007*
Inferior parietal gyrus	L	2.193 ± 0.066	2.093 ± 0.078	<0.001*
Supramarginal gyrus	L	2.111 ± 0.054	2.044 ± 0.062	<0.001*
Angular gyrus	L	2.123 ± 0.073	2.000 ± 0.09	<0.001*
Precuneus	L	2.212 ± 0.033	2.169 ± 0.049	<0.001*
	R	2.137 ± 0.04	2.100 ± 0.044	<0.001*
*Occipital lobe*				
Calcarine fissure and surrounding cortex	L	2.249 ± 0.039	2.217 ± 0.038	<0.001*
Cuneus	L	2.129 ± 0.038	2.109 ± 0.043	0.013*
Lingual gyrus	L	2.202 ± 0.038	2.168 ± 0.045	<0.001*
Superior occipital gyrus	L	1.950 ± 0.064	1.891 ± 0.076	<0.001*
Middle occipital gyrus	L	2.191 ± 0.045	2.124 ± 0.068	<0.001*
*Temporal lobe*				
Superior temporal gyrus	L	2.181 ± 0.051	2.120 ± 0.053	<0.001*
Middle temporal gyrus	L	2.336 ± 0.034	2.303 ± 0.048	<0.001*
*Limbic system*				
Posterior cingulate gyrus	L	1.989 ± 0.054	1.945 ± 0.05	<0.001*
Parahippocampal gyrus	R	2.142 ± 0.03	2.157 ± 0.031	0.011*
*Subcortical regions*				
Amygdala	R	1.944 ± 0.04	1.968 ± 0.042	0.005*
Caudate nucleus	L	2.075 ± 0.047	2.051 ± 0.047	0.010*
	R	2.088 ± 0.04	2.044 ± 0.046	<0.001*
Lenticular nucleus, putamen	L	2.105 ± 0.075	2.066 ± 0.049	0.003*

*Significant difference under a corrected threshold of FDR = 0.05

Std: standard deviation

### The progression rates of cortical atrophy in the supratentorial regions were similar

**Fig 7:**
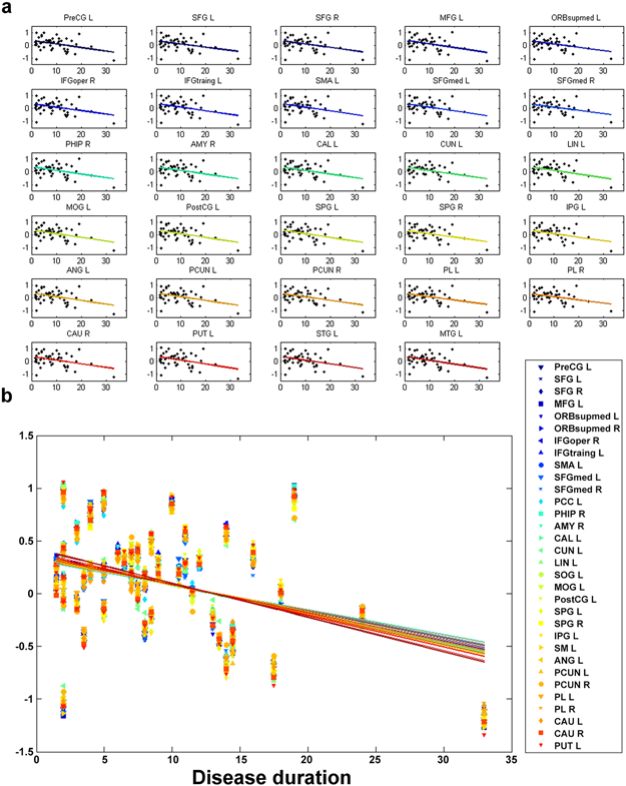
The progression rates of cortical atrophy in the supratentorial regions were similar. (a) Excluding the age effect, the 3D-FD values of 32 cerebral regions had significant negative correlations with disease duration. (b) No significant difference was observed among these individual regions using ANOVA with Bonferroni post-hoc analysis (Degrees of freedom for regions = 98 and F = 0.02).

In SCA3 patients, we observed a significant correlation (*p* < 0.05) between disease duration and the 3D-FD values of 37 specific regions, including the anterior and posterior lobes of the cerebellar cortex and frontal lobe, parietal lobe, occipital lobe, temporal lobe, and limbic and subcortical regions of the cerebral cortex. To exclude the effect of age, we conducted multiple regression analysis to verify the association between disease duration (as an independent variable) and 3D-FD values (as a dependent variable). The results indicated that all 37 regions exhibited significant correlation with disease duration when the effect of age was excluded (Fig 7a). In addition, we observed similar progressions of the decreasing 3D-FD value in 32 cerebral regions as the disease duration increased (Fig 7b). No significant difference was observed among these individual regions by using an ANOVA with Bonferroni post-hoc analysis. The degrees of freedom for regions = 98, and F = 0.02.

## Discussion

### Cortical change was correlated with cerebellar dysfunction in SCA3

In this study, decreased cortical complexity in cerebellum of SCA3 inferred the cortical atrophies in cerebellum. Similar morphological changes in the cerebellum of the SCA3 patients were reported by previous studies using MRI-based volumetric methods [52, 53]. Furthermore, the 3D-FD value in the cerebellar cortex of the SCA3 patients exhibited a significant negative correlation with SARA scores, which demonstrated that the 3D-FD method is effective in quantifying cortical changes and evaluating the association between cortical complexity and dysfunction of the cerebellar cortex in SCA3 patients.

### SCA3 patients exhibited a widespread atrophic area in the supratentorial region

Cerebellar cortex, especially the posterior lobe, shows significant decrease in SCA3 patients, and indicated the motor-related impairments in SCA3. Nonetheless, when pathology is in the lateral hemisphere of the posterior cerebellum, the universal cerebellar impairment manifests the cerebellar cognitive affective syndrome (CCAS), including motor-related impairments, executive and visual-spatial impairments, and with affective disturbance [54–56]. Previous studies indicated that the clinical features of SCA3 are in accordance with the CCAS [57, 58]. Moreover, the involvement of posterior cerebellum was supported by previous pathological studies, which indicated that the middle cerebellar peduncle and central white matter of the cerebellum of SCA3 patients were primarily affected, and the cerebellar cortex and Purkinje were mildly affected [59]. However, the involvement of the posterior lobe of the cerebellar cortex in SCA3 patients has not been observed in previous VBM studies [14, 16, 60]. Unlike these VBM studies, the results of this study indicated that the cerebellar lobes, including the posterior lobe, are extensively involved in SCA3 patients. These findings are consistent with pathological findings related to SCA3 patients and support the hypothesis that the clinical features in SCA3 were consistent with CCAS [54].

The CCAS results from the disruption of the corticocerebellar system [54, 55, 61], which is associated with the prefrontal, superior parietal, superior temporal, and limbic cortices [51, 62]. Patients with CCAS exhibited impairments in executive functions, shifting, verbal fluency, abstract reasoning, and working memory, all of which are associated with visuospatial deficits, personality changes, and language deficits [51, 62]. These cognitive and emotional impairments imply the presence of widespread extracerebellar lesions in the cerebral cortex [63–67]. Our results indicated a significant difference in the supratentorial regions of SCA3 patients, including the frontal, parietal, temporal, occipital, and limbic lobes and subcortical regions. Moreover, a significant negative correlation was observed between the 3D-FD values of these individual regions and disease duration in SCA3 patients. This result revealed that the involvement in SCA3 patients was more widespread than the traditional olivopontocerebellar atrophy pattern [68] and the corticocerebellar system.

### FD changes of the cerebral cortex explain why the cerebral-cortex-related mental decline of SCA3 patients is apparent at the late stage of the disease

Our results suggested that cerebral abnormality is more apparent at the late stage of SCA3 because the mean 3D-FD values at the early stage were greater than the late stage. This result is consistent with the presence of dementia at the late stage of SCA3 [69]. Conversely, the MMSE scores show no statistical difference between SCA3 patients and normal controls throughout the disease duration.

Moreover, the observations of abnormal EEG signals in the cerebral hemispheres can provide additional information in supporting cerebral involvement, such as abnormal activities in frontal and temporal regions of schizophrenics [70–72] and in parietal and occipital lobe of affective disorders [71, 73]. Our results demonstrated abnormal EEG recordings in part of SCA3 patients, namely, intermittent slowing and diffuse background slowing, which were in line with previous SCA3 studies [69, 74]. Both the background slowing and slow-wave activity may result from the clinical entities involving cortical regions [75, 76]. Besides, EEG signals reflect only superficial cortical activity, these results can be considered direct physiological evidence supporting cortical involvement in SCA3 patients. Moreover, the duration of SCA3 in patients with abnormal EEG signals has the trend to be longer than that in patients with normal EEG signals. This result is consistent with those of previous studies indicating that abnormal EEG signals in SCA3 patients are observed primarily at the late stage of the disease [69, 77]. Our results suggested involvement of the cerebral cortex in SCA3 patients throughout the disease duration; however, abnormally neuronal viability of the cerebral cortex of SCA3 patients can be detected at the late stage of the disease using EEG.

In this study, we demonstrated the feasibility of detectible changes of cortical complexity in SCA3 patients. Our results indicate that 3D-FD is the most sensitive measurement for SCA3 in detecting the cerebral cortical involvement and mental associated deficits, especially in early stage of disease duration. Because of limited explanatory power given by the small sample size in EEG recording and MMSE scores of SCA3, we only concluded that (1) the changes of cortical complexity in SCA3 are more substantial than the changes of EEG recording and MMSE scores in SCA3; (2) the cortical involvement in SCA3 patients can be supported by abnormally neuronal viability of the cerebral cortex in SCA3 patients. Besides, our results regarding EEG recording and MMSE scores were in line with previous studies [69, 74, 78, 79].

Another limitation might be the sensitivity of MMSE in detecting the frontal-executive and subcortical dysfunction [80]. However, the MMSE has satisfactory reliability, internal consistency and test–retest reliability. Considering some SCA3 patients were difficult to tolerate a long time examination, MMSE requires only 5 to 10 minutes for setting up and is an easy tool for screening the cognitive impairments. Other specific assessments of cognitive abilities and related mental deficits need further be conducted. It would be helpful to investigate the correlation between the cognitive impairments in SCA3 patients and the changes of cortical complexity of SCA3 patients.

In conclusion, the SCA3 patients in this study exhibited reduced complexity in specific supratentorial regions, which were more extensive than traditional olivopontocerebellar regions and the corticocerebellar system. The cortical atrophy quantified using the 3D-FD method was observed in early-stage SCA3 patients, who exhibited no decreased MMSE scores or abnormal EEG patterns. In addition, cortical complexity changes were more apparent at the late stage of SCA3, indicating that abnormal EEG signals are observed only at the late stage of the disease. Therefore, we suggest that the 3D-FD method is more suitable for detecting cortical atrophy early in SCA3 patients than conventional measurements.

### Ethical standards

This study was conducted in accordance with the Declaration of Helsinki and was approved by the Institutional Review Board of Taipei Veterans General Hospital. All participants provided written informed consent before participating in this study.
